# Social Problems of the Day

**Published:** 1907-09-28

**Authors:** 


### SOCIAL PROBLEMS OF THE DAY.

### THE STATE CHILDREN'S AID ASSOCIATION.

THE sixth report of the State Children's Aid Association, comprising the work done in the years 1904, 1905, and 1906, is moderately encouraging to those who believe that it is best for the welfare of pauper children that they should be brought up in conditions as nearly as possible resembling those of a good normal home. There is but a small increase in the number of children boarded out either beyond or within the union to which they belong—and it is to boarding out beyond that one must look for the eradication of evil influences and the pauper taint; but there is an increase in the number of unions which are now placing the children under their care in scattered homes instead of in barrack schools. The scattered home has the advantage, in the eyes of Guardians, that it is more easily inspected and more completely under Poor-law control than the distant cottage where one or two children are boarded out. The inspection difficulty is the greatest in boarding out. The Guardians must to some extent be guided by the advice of the Local Committee, and occasionally the members of that, in choosing homes for the children of the State, are more influenced by a desire to provide for their own dependents than by a careful determination to place the children in the best possible environment. Another difficulty is the jealousy of those who are not trusted with the union children against those who receive them, and it is unfortunate that this jealousy is echoed by those who have no personal grievance in the matter, and who have advantages of education and position that should enable them to take a wider and more philanthropic view of the question. For our own part, we believe that the upbringing to be obtained in any respectable cottage is the best for a child; but Guardians require to be assured of the absolute single-mindedness of those to whom they must necessarily leave great part of the supervision of any child boarded outside the union. Lastly, there are always to be found in every Board of Guardians members who, in the supposed interests of economy, protest against allowing the expenses of a Guardian or Guardians who are sent to see a single child or a handful of children who are boarded at any considerable distance. Yet such visits are necessary, and we doubt if the utmost sum that was ever spent in such travelling expenses would make up the difference in cost between the maintenance of a boarded-out child and that of one in a barrack school. But this is a point which these short-sighted economists overlook.

Scattered homes are a fairly good compromise between boarding-out and herding in barracks. The placing of ten or twelve children of different ages under the care of a good kindly woman fairly well simulates home conditions, and, as the children go to the ordinary school and mix in other ways with the mass of the population, they get that training in citizenship which is the most valuable part of education; but it is to be feared that in any, even the humblest, community the scattered home may suffer a kind of segregation which will make the children feel that they are paupers, and nullify the intentions of those who removed them from the workhouse and its environments. But such a criticism, if applicable to the scattered home, is far more true of the self-centred village community. In these, children drawn from the same environment form a little world of their own, with which the outer world comes only slightly into contact. The system is, moreover exceedingly costly. The village community of the Bermondsey Guardians, at Shirley, accommodating 560 children, cost over £320 per bed, while the average cost of each child maintained there was £1 1s. 9d. a week. Poplar lived up to its reputation by spending £162,000 in providing accommodation for about 700 children. This expense is not at[illegible]ed for by any special advantage in the community system. As the children are housed in numbers of from forty to sixty in one building the whole tone of the place must be more institutional than home-like. The supervision may be more individual and thorough than in a barrack school, but otherwise the conditions are no better. Infectious disease, when once introduced among a large number of children is difficult to eliminate and has a smaller field to conquer there than in the larger schools; but at the best the difference between the village community and the barrack school is one more of degree than of kind.

The report mentions two of the defects of barrack schools, in which 11,809 of the 68,000 children who are supported out of the rates are at present living. One of these is the liability to infectious disease, of which we have spoken, and which is attested by the reports of medical officers and superintendents of these institutions. The other, according to the statements of H.M. Inspectors of Schools, is that the standard of education is lower than in ordinary elementary schools. Another fault is that the children grow up in far more comfort than they are likely to meet with in the world without. Pauperism has been made pleasant to them in their youth, and they will not make any great struggle against it in their adult life.

Among the other work to which the State Children's Association gives its attention is the promotion of special Courts of Justice for children. At present there is only one special separate Court for juvenile offenders—that established at Birmingham through the energy of Mr. Courtenay Lord, but in many large towns children's cases are now heard separately. A consequence of the establishment of Children's Courts must be the appointment of probation officers, to whose care the children are committed, instead of being sent to industrial schools. He visits them in their homes, reports on their conduct, and assists and befriends them as he can. The Association is also agitating for that very necessary amendment of the Infant Life Protection Act, which will make it necessary to register, and possible to inspect, the "one-nurse child" cases among whom mortality due—one may suspect, but cannot prove—to neglect, if not to foul play, is so common. The Association is also using its influence in support of a Bill for the better protection of the children of vagrants, whom they would fain save from following in the ways of their parents. Indeed, with modest but persistent effort, this Association is helping every influence that promises to aid these children, so sorely handicapped in the race of life, to grow up into healthy, intelligent, self-respecting citizens. The offices of the Association are at 58 Old Broad Street, E.C. The Chairman is Lord Burghclere, and the Hon. Secretary Mrs. S. A. Barnett.

